# Flash Pulmonary Edema Triggered by Tadalafil Use in a Patient With Chronic Left Bundle Branch Block

**DOI:** 10.7759/cureus.92534

**Published:** 2025-09-17

**Authors:** Renat Roytenberg, Oscar Rodrigo Zamudio Herrera

**Affiliations:** 1 Department of Cardiology, Marshall University Joan C. Edwards School of Medicine, Huntington, USA

**Keywords:** erectile dysfunction, flash pulmonary edema, left bundle branch block (lbbb), pde5 inhibitors, uncontrolled hypertension

## Abstract

This case report discusses the diagnosis and management of flash pulmonary edema (FPE) in a 61-year-old male patient with a history of uncontrolled hypertension, type 2 diabetes, dyslipidemia, erectile dysfunction, and chronic complete left bundle branch block. The patient presented with acute respiratory distress two hours after tadalafil use. Evaluation revealed hypoxemia and diffuse pulmonary edema, with echocardiography revealing normal systolic function and tissue Doppler imaging suggesting grade I diastolic dysfunction. The patient’s symptoms resolved with oxygen, diuresis, and discontinuation of tadalafil. This case emphasizes the need for caution when prescribing phosphodiesterase type 5 inhibitors such as tadalafil to patients with structural or conduction cardiac abnormalities and the importance of prompt recognition and management of FPE in this population.

## Introduction

Flash pulmonary edema (FPE) is a distinct and often life-threatening form of pulmonary edema defined as a dramatic form of acute decompensated heart failure [1], where an acute and abrupt fluid accumulation in the lung parenchyma occurs, which develops within minutes to hours [2]. FPE is commonly associated with bilateral renal artery stenosis [3,4] and hypertension [5] and has been documented in cases of rate-related left bundle branch block (LBBB) [6,7]. In-hospital mortality rates for patients presenting with acute pulmonary edema range drastically, from 12% [8] to 46% [9], for example, but typically include patient populations with acute coronary syndrome (ACS) or heart disease. However, one study determined a mortality rate of 31.5% [10] due to acute pulmonary edema in non-ACS patients where 40% of cases included chronic atrial fibrillation and 36% showed previous heart failure. Regardless, data on in-hospital mortality of FPE in patients without overt heart disease or ACS have not been previously described.

Nitric oxide (NO) is released from several cell types, including endothelial cells and nerve cells, upon cell signals such as sheer stress, and acts to activate soluble guanylyl cyclase to generate cyclic guanosine monophosphate (cGMP) [11]. cGMP activates protein kinase G, which leads to the relaxation of vascular smooth muscle via deactivating myosin light chain kinase in a calcium-dependent manner [11]. Phosphodiesterases hydrolyze cGMP, and phosphodiesterase type 5 (PDE5), which is expressed especially in pulmonary vasculature and the corpus cavernosum [12], can be targeted by PDE5 inhibitors to treat erectile dysfunction and pulmonary arterial hypertension (PAH) [13,14]. However, the occurrence of FPE in the context of PDE5 inhibitor use, such as tadalafil, remains largely unexplored. While sildenafil, another PDE5 inhibitor, has been linked to cases of pulmonary edema [15], there are no PubMed-indexed case reports, case series, or pharmacovigilance data on PDE5 inhibitor-induced FPE. The incidence of FPE associated with PDE5 inhibitors is likely rare, as incidence or association rates have not been described in the literature.

Predisposing conditions, including hypertensive emergencies, left heart dysfunction, and structural cardiac abnormalities such as chronic complete LBBB, may amplify the risk of developing FPE. LBBB is a conduction disorder in which electrical impulses are delayed or fail to propagate through the left bundle branch of the His-Purkinje system. Consequently, activation of the left ventricle occurs later than that of the right ventricle, leading to ventricular dyssynchrony. LBBB is associated with systolic and/or diastolic dysfunction, which may be caused by ventricular remodeling triggered by asynchronous myocardial activation [16]. Rate-related LBBB is a functional conduction abnormality rather than a fixed structural defect in the left bundle branch and arises when the heart rate exceeds a certain threshold, resolving when it moves below the threshold [6,7].

FPE and postcoital hemoptysis have been observed in a patient with mitral regurgitation [17]. A case series has documented pulmonary edema in two patients post-sexual intercourse, including a case involving transient T wave inversions on electrocardiogram (EKG) and another case involving longstanding hypertension and left ventricular dilation [18], though the exact temporal associations with pulmonary edema were not described.

Consequently, the vasodilatory effects of tadalafil can increase pulmonary capillary pressure (PCWP) in susceptible individuals, exacerbating pulmonary venous congestion and triggering FPE. This report, for the first time, details a case of FPE triggered by tadalafil use in a patient with uncontrolled hypertension and chronic LBBB, emphasizing the interplay of these factors in the pathophysiology of this rare adverse event.

## Case presentation

A 61-year-old male patient with a history of uncontrolled hypertension, type 2 diabetes mellitus, dyslipidemia, erectile dysfunction, and chronic LBBB presented to the emergency department (ED) with acute respiratory distress. The patient reported engaging in sexual intercourse approximately two hours after taking tadalafil. During intercourse, he experienced sudden-onset shortness of breath, a gurgling sensation in his chest, and a productive cough with frothy, pink sputum. His wife, a nurse, measured his blood pressure at 220/80 mm Hg, with an oxygen saturation below 90%. The patient did not have chest pain. The patient denied a history of dyspnea or angina on exertion. He had no history of exercise limitations due to chronic LBBB. The patient had no history of any similar episode in the past. Home medications included simvastatin, metformin, lisinopril, levothyroxine, atenolol, aspirin, and tadalafil.

On arrival at the ED, vital signs showed hypertensive emergency (197/76 mm Hg) and hypoxemia (oxygen saturation: 86%; PaO₂: 53 mm Hg). Pulse was 82 bpm, and respiratory rate was 18 breaths/min. Physical examination revealed increased work of breathing and diffuse rhonchi. Laboratory findings are shown in Table 1.

**Table 1:** Laboratory findings on presentation. DM: diabetes mellitus; PaO₂: arterial partial pressure of oxygen

Lab test	Value	Units	Reference range
Troponin (high sensitivity)	11 (0 hrs), 14 (2 hrs), 15 (6 hrs)	ng/mL	<14 ng/mL
Brain natriuretic peptide (BNP)	103	pg/mL	<100 pg/mL
PaO₂	53	mm Hg	75-100 mm Hg
Creatinine	1.1	mg/dL	0.6-1.2 mg/dL
Hemoglobin A1c (HbA1c)	6.8	%	<5.7% (normal), 5.7-6.4% (pre-DM), ≥6.5% (DM)
Low-density lipoprotein (LDL)	52	mg/dL	<100 mg/dL

**Figure 1:**
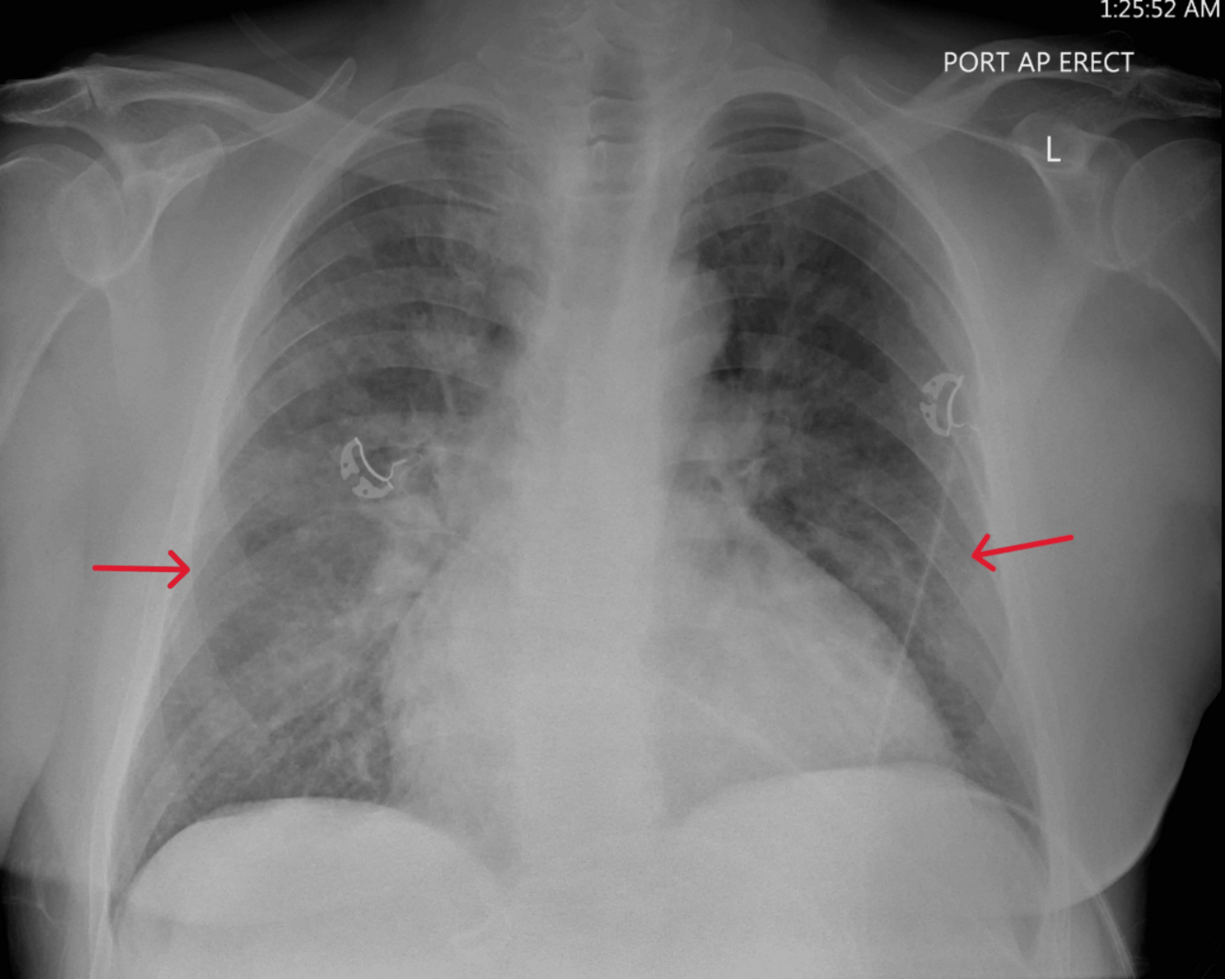
CXR (anteroposterior) demonstrates signs of pulmonary edema. Red arrows indicate increased bilateral opacities signifying pulmonary edema. CXR: chest x-ray

**Figure 2:**
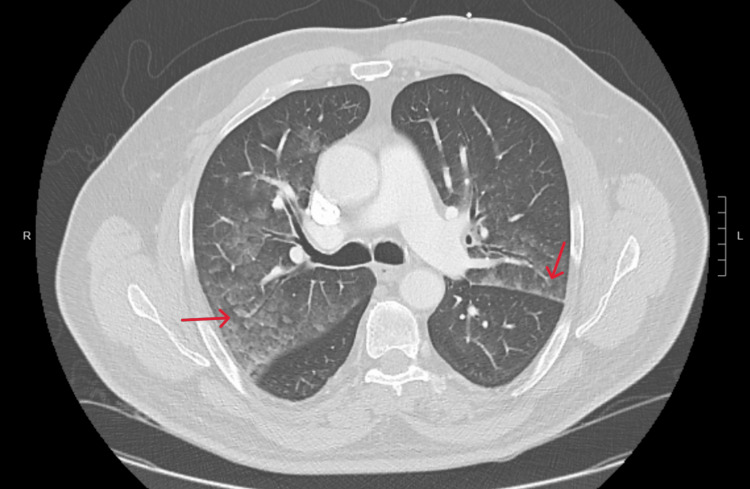
CT (axial plane) shows pulmonary edema. Red arrows indicate bilateral hazy increased attenuation signifying pulmonary edema. CT: computed tomography

**Figure 3:**
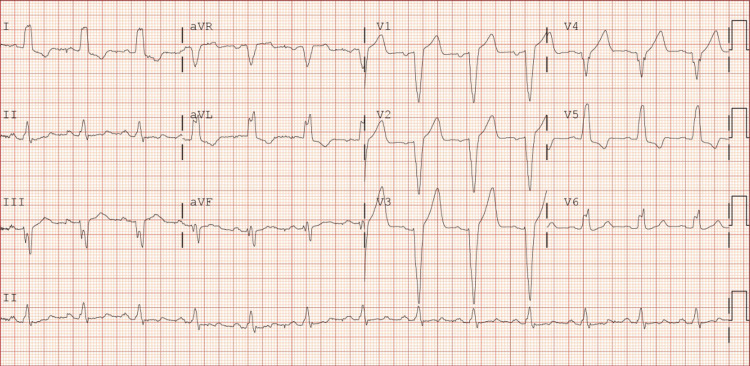
Electrocardiogram indicating sinus rhythm with complete left bundle branch block (LBBB). Rate: 78 bpm. LBBB findings include broad, notched R waves in the lateral leads (I, aVL, V6); deep, wide S waves in the right precordial leads (V1–V3); absent Q waves in the lateral leads (I, aVL, V5-V6); prolonged QRS duration (>120 ms).

Chest x-ray (CXR) (Figure 1) and computed tomography (CT) (Figure 2) were consistent with pulmonary edema. Imaging showed no signs of fibrosis, consolidation, or pulmonary embolism. EKG showed sinus rhythm with complete LBBB with some premature ventricular contractions (PVCs) (Figure 3). Echocardiography revealed normal global systolic left ventricular function (ejection fraction: 50%-55%), normal right ventricular systolic function (tricuspid annular plane systolic excursion: 2.3 cm), and no valvular abnormalities. Tissue Doppler indices, including E/A ratio, suggested a pattern consistent with grade I diastolic dysfunction (Table 2). Additionally, renal artery Doppler ultrasonography demonstrated peak systolic velocities, renal-to-aortic ratios, and resistive indices within normal limits, consistent with no evidence of renal artery stenosis.

**Table 2:** Tissue Doppler indices E/A ratio: ratio of early (E) to late (A) transmitral inflow velocities, with values < 1 indicating impaired relaxation and >2 suggesting restrictive filling; e′ medial (septal) velocity: early diastolic mitral annular velocity at the septal (medial) annulus, reflects LV relaxation; e′ lateral (lateral annular) velocity: early diastolic mitral annular velocity at the lateral annulus, reflects LV relaxation; E/e′ medial (septal) ratio: ratio of mitral inflow E velocity to septal e′ velocity, estimates LV filling pressure; E/e′ lateral ratio: ratio of mitral inflow E velocity to lateral e′ velocity, estimates LV filling pressure; LAVI: left atrial volume index, reflects chronic LV filling pressures; TR velocity: tricuspid regurgitation peak velocity, used to estimate pulmonary artery systolic pressure; LV: left ventricular *Per the American Society of Echocardiography (ASE) and the European Association of Cardiovascular Imaging (EACVI) guidelines [19]

Tissue Doppler index	Value	Units	Reference range^*^
E/A ratio	0.6		1.0-2.0
e' medial	4.57	cm/s	≥7 cm/s
e' lateral	6.85	cm/s	≥10 cm/s
E/e' medial ratio	12.4		≤8 (abnormal if >15)
E/e' lateral ratio	8.3		≤8 (abnormal if >12)
LAVI	27.3	mL/m²	≤34 mL/m²
TR velocity	1	m/s	≤2.8 m/s

The patient was treated with oxygen supplementation and intravenous furosemide (40 mg, twice daily), and tadalafil was discontinued. His symptoms resolved in 48 hours with this management. He had no further events for the rest of his hospitalization.

## Discussion

This case highlights the potential for tadalafil to precipitate FPE in susceptible individuals. FPE is a multifactorial condition, and in this case, it was challenging to determine whether tadalafil use, underlying cardiovascular disease (uncontrolled hypertension and chronic LBBB), or physical activity was the primary trigger. Post-coital pulmonary edema is extremely rare and has been documented in three cases featuring distinct cardiac pathology compared to our case, including mitral regurgitation [17], ventricular dilation [18], and T wave inversions on EKG [18]. The mildly elevated troponin levels in the context of the absence of chest pain and any EKG changes in this case suggest that ACS is not likely to explain FPE in this patient. Similarly, the mildly elevated BNP level and normal echocardiogram likely reflected a transient increase in ventricular wall stress and filling pressures, consistent with a hemodynamic profile of pulmonary edema.

The patient’s clinical picture strongly supports a transient surge in left-sided filling pressures. The combination of enhanced preload due to tadalafil-induced venodilation, abrupt afterload increase from hypertensive emergency, and potentially impaired ventricular coordination from chronic LBBB likely contributed to acute pulmonary venous congestion. LBBB is known to impair ventricular filling and ejection efficiency [20], potentially elevating PCWP in response to increased preload or afterload. The rapid resolution of symptoms with diuresis and oxygen supports a hemodynamic etiology consistent with FPE.

Tissue Doppler imaging revealed a low E/A ratio, reduced e' velocities, and borderline E/e' ratios, suggesting a pattern consistent with grade I diastolic dysfunction. In grade I diastolic dysfunction, resting PCWP is often normal, but PDE5 inhibitors can tip the balance by shortening filling times. This may cause transient rises in PCWP and increase the risk of pulmonary edema. Right heart catheterization also would have been a useful method to collect meaningful hemodynamic measurements but was relatively contraindicated due to the patient's hypoxemia and hypertensive emergency. Additionally, we do not believe that occasional PVCs would have affected diastolic filling time. Therefore, we suggest that the patient’s threshold to develop FPE may have been lowered by chronic LBBB, grade I diastolic dysfunction, and hypertensive emergency and that tadalafil usage triggered FPE.

To the best of our knowledge, current literature lacks data on tadalafil-induced pulmonary edema. However, sildenafil, with a shorter half-life (4-5 hours) compared to tadalafil (17.5 hours) [14], has been implicated in pulmonary edema in 2/22 patients in a retrospective pilot study of pediatric patients with Group 2 pulmonary hypertension. The pediatric patients in this study also had concomitant left-sided heart failure [15]. Pulmonary edema subsided after discontinuation of sildenafil in this patient population.

This case highlights the need for caution when prescribing PDE5 inhibitors in patients with predisposing cardiovascular conditions that can contribute to increasing left ventricular end-diastolic pressure [3] or PCWP, such as LBBB and uncontrolled hypertension. We recommend a multifactorial risk assessment in patients with complex cardiovascular histories requesting PDE5 inhibitors, including baseline echocardiography, baseline EKG, and evaluation of blood pressure control. Furthermore, in rare but serious adverse events such as in this case, reporting to regulatory agencies (e.g., FDA MedWatch) or directly to the manufacturer is a critical step for pharmacovigilance and may uncover safety signals that were not apparent during pre-approval trials.

## Conclusions

This case highlights the need to exercise caution when prescribing PDE5 inhibitors, particularly for patients with conduction or structural cardiac abnormalities and/or longstanding hypertension. Pulmonary vasodilation caused by a PDE5 inhibitor in a setting that predisposes to elevated PCWP or left heart filling pressures can flood the precapillary vasculature and lead to pulmonary edema. Clinicians should be mindful of the potential for FPE and consider alternative treatments for erectile dysfunction in high-risk populations.
